# First experiences with automated annular suturing device in totally endoscopic aortic and mitral valve replacement

**DOI:** 10.1093/icvts/ivae112

**Published:** 2024-06-03

**Authors:** Ali El-Sayed Ahmad, Saad Salamate, Nermir Granov, Ali Bayram, Sami Sirat, Mirko Doss, Miriam Silaschi, Ömür Akhavuz, Farhad Bakhtiary

**Affiliations:** Department of Cardiac Surgery, University Hospital Bonn, Bonn, Germany; Department of Cardiac Surgery, University Hospital Bonn, Bonn, Germany; Department of Cardiac Surgery, Clinical Center University of Sarajevo, Bosnia and Herzegovina; Division of Cardiac Surgery, Heart Centre Siegburg, Siegburg, Germany; Division of Cardiac Surgery, Heart Centre Siegburg, Siegburg, Germany; Division of Cardiac Surgery, Heart Centre Siegburg, Siegburg, Germany; Department of Cardiac Surgery, University Hospital Bonn, Bonn, Germany; Department of Cardiac Surgery, University Hospital Bonn, Bonn, Germany; Department of Cardiac Surgery, University Hospital Bonn, Bonn, Germany

**Keywords:** Minimally invasive valve surgery, Mitral valve, Right anterior mini-thoracotomy device, Aortic valve, Automated annular suturing device

## Abstract

**OBJECTIVES:**

To overcome some of the challenges of endoscopic minimally invasive valve surgery, an automated annular suturing device has been used in aortic and mitral valve replacement surgeries. The current study investigates early clinical outcomes of patients who received aortic or mitral valve replacement with the help of the RAM^®^ device as first experiences in minimally invasive valve surgery.

**METHODS:**

Between September 2020 and June 2023, 66 consecutive patients (mean age 61.8 ± 11 years) underwent endoscopic minimally invasive aortic or mitral valve replacement through right anterior mini-thoracotomy at 2 cardiac surgery referral centres in Germany. The RAM^®^ device was used in all Patients. 3.5 and 5.0 sizes were used in 16.7% and 83.3% of patients, respectively. Aortic, mitral and double valve surgery was performed in 81.8%, 15.2% and 1.5% of patients, respectively. Clinical data were prospectively entered into our institutional database.

**RESULTS:**

Cardiopulmonary bypass time and cross-clamping time were 97.9 ± 20.9 and 66 ± 15.7 min, respectively. Intensive care unit and hospital stays were 1 [1–2] and 9 [7–13] days, respectively. No paravalvular leak and no other intraoperative complications occurred. 30-day and in-hospital mortality were zero. Conversion to sternotomy occurred in 1 (1.5%) patient due to bleeding.

**CONCLUSIONS:**

The usage of the RAM^®^ device is a safe, feasible and effective approach to the endoscopic implantation of aortic or mitral valves and yield excellent early outcomes. Larger size studies are needed to evaluate the efficacy and safety of RAM^®^ device.

**Figure:**
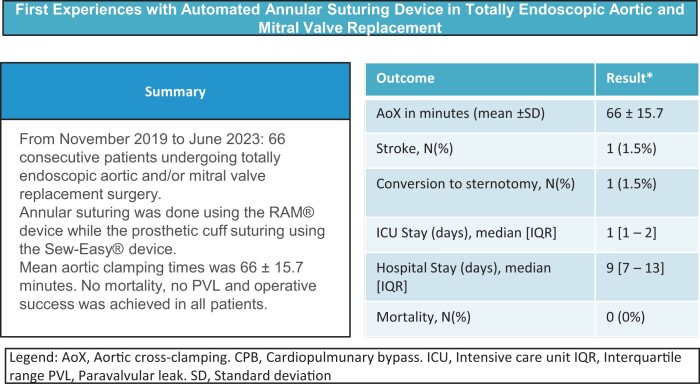


## INTRODUCTION

In recent years, minimally invasive valve surgery (MIVS) has gained popularity in cardiac surgery centres across the globe. This innovative approach has been shown to be a safe and effective alternative to traditional surgical methods in various studies while offering some favourable outcomes relating to a reduced surgical trauma and pain, blood loss, shorter intensive care unit (ICU) and hospital stay, amongst others [1–3]. Among the many techniques that fall under MIVS, right anterior mini-thoracotomy (RAMT) has emerged as a promising approach. RAMT has been made possible due to advancements in technologies for cardiopulmonary bypass (CPB), specialized surgical and interventional instruments and thoracoscopy. Nevertheless, the technique is demanding with a sharp learning curve and entails numerous technical obstacles. Among them is the task of positioning sutures in the valvular annulus within a restricted field of view and space [4].

To address this challenge, the RAM^®^ device (LSI Solutions^®^, based in Victor, NY, USA) was used in our institution to facilitate the annular suturing for MIVS through RAMT. The purpose of this study is to evaluate the early clinical outcomes, safety, feasibility and efficacy of minimally invasive aortic or mitral valve replacement procedures using the RAM^®^ device. This study aims to provide insights into the use of RAM^®^ as a first experience in MIVS.

## MATERIALS AND METHODS

Between November 2019 and June 2023, 66 patients (mean age 61.8 ± 11 years, body mass index 26.7 ± 4.7) underwent endoscopic MIVS through RAMT at 2 cardiac surgery referral centres in Germany (Heart Centre Siegburg/Heart Centre Bonn). Demographics, clinical and procedural data were prospectively collected and were documented in the dedicated database at our institutions. The study was approved by the respective institutional review boards (Medical Association of North Rhine number 82/2021, local ethics board of the University of Bonn number AZ184/23-EP). Individual patient consent for the study was waived.

Arterial hypertension, diabetes mellitus and coronary artery disease were represented in 57.6%, 15.2% and 43.9% of patients, respectively. Baseline characteristics of the study population are shown in Table 1.

**Table 1:** Patient characteristics

Characteristic (total *N* = 66)	*N* (%)
Female	23 (34.8%)
Age (years), mean ± SD	61.8 ± 11
BMI (kg/m^2^), mean ± SD	26.7 ± 4.7
Euroscore II, median [IQR]	1.01 [0.84–1.55]
Diabetes	10 (15.2%)
EF Range	
≥50	56 (84.4%)
31–50	9 (13.6%)
21–30	1 (1.5%)
Arterial hypertension	38 (57.6%)
CAD	29 (43.9%)
1 vessel disease	22 (33.3%)
2 vessel disease	3 (4.5%)
3 vessel disease	4 (6.1%)
Previous heart surgery	2 (3%)
Aortic stenosis	44 (66.7%)
Aortic insufficiency	35 (53%)
Bicuspid valve	23 (34.8%)
Aortic calcification	49 (74.2%)
Mitral stenosis	3 (4.5%)
Mitral insufficiency	16 (24.2%)
Renal failure	4 (6.1%)
NYHA	
I	4 (6.1%)
II	8 (12.1%)
III	54 (81.8%)
Endocarditis	0 (0%)

All values are represented as *N* (%) unless otherwise specified.

BMI: body mass index; EF: ejection fraction; NYHA: New York Heart Association classification.

**Figure 1:**
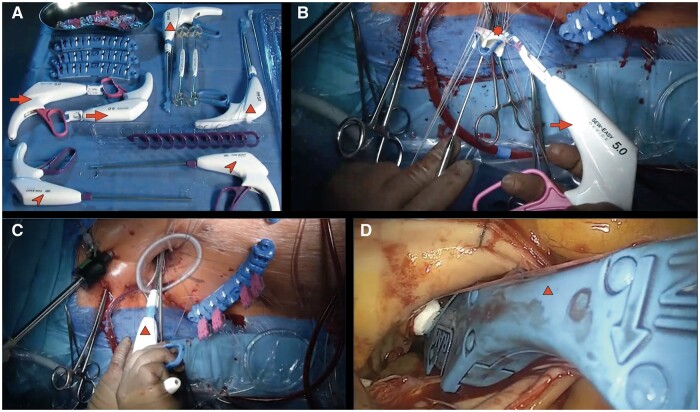
(**A**) Operating tray showing 2 RAM^®^ devices (triangle), 2 Sew-Easy^®^ devices (arrows) and 2 Cor-Knot devices (arrow heads). (**B**) Sew-Easy^®^ (arrow) device being used to insert the sutures into the prosthetic valve cuff (star). (**C**) External view of the RAM^®^ device in use. (**D**) Endoscopic view of the RAM^®^ device in use.

**Figure 2:**
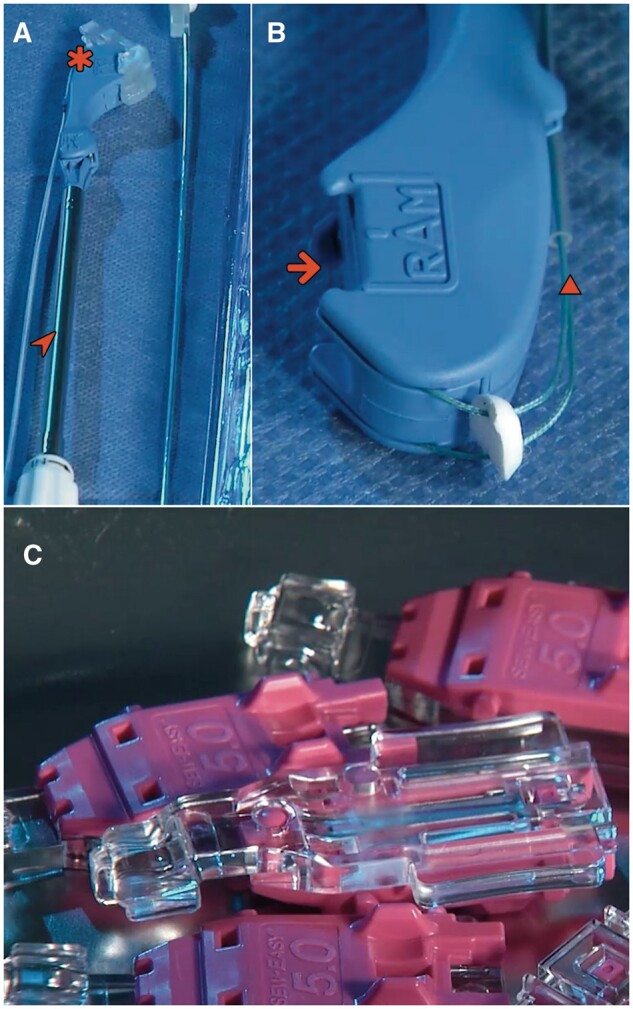
(**A**) Shaft (arrowhead) and tip of the RAM^®^ device. RAM^®^ Cor-Suture Quick Load unit is attached to the tip of the device (asterisk). (**B**) Tip of the RAM^®^ device with visible tissue jaw (arrow) and an installed Cor-Suture ( triangle) and (**C**) Sew-Easy^®^ Cassette.

The RAM^®^ device is an innovative surgical tool designed to assist in the suturing process by enabling the placement of horizontal mattress stitches using dual curved needles. The device is available in 2 sizes, 3.5 and 5 mm, based on the required suture spacing. It consists of a handle, a lever and a long-articulated shaft that terminates with the tip of the device (Figs 1 and 2).

The detailed description of the RAM^®^ device and its use were previously published [5]. 3.5- and 5.0-mm sizes were used in 11 (16.7%) and 55 (83.3%) patients, respectively, in accordance to the respective valvular ring size; roughly translating to 3.5-mm-sized bites for 21-mm prostheses and the 5-mm size for valves of larger diameter. Patient selection for the use of the RAM^®^ device was based on surgeon preference and experience with the device itself. While all the procedures were performed by a team of cardiac surgeons experienced in minimally invasive techniques, the RAM^®^ device was not introduced in each of their individual standard approach at the same time and as such was not employed in all consecutive patients undergoing endoscopic aortic and/or mitral valve replacement surgery. Additionally, it was not used in patients with smaller valvular ring sizes, such as lower than 21 mm due to size constraints with regard to the horizontal extent of the annular mattress sutures.

The primary end-point of this study was operation-related mortality, which was defined as in-hospital mortality occurring within 30 postoperative days. Secondary end-points were technical success, CPB and aortic cross-clamping (AoX) times, paravalvular leak (PVL), number of packed red blood cells transfused, in-hospital stay and ICU duration, intrathoracic bleeding requiring surgery, and in-hospital morbidity.

The latter included stroke, delirium, acute kidney injury, new-onset atrial fibrillation, atrioventricular block requiring a temporary pacemaker (PM), low cardiac output syndrome, number of procedure-related wound revision, and respiratory insufficiency requiring invasive management, namely prolonged intubation (≥24 h) and tracheostomy.

### Operative techniques

Apart from the use of the RAM^®^ device, our step-by-step approach to endoscopic minimally invasive aortic valve replacement through RAMT was previously published [6].

Conventional general anaesthesia and double-lumen lung ventilation are used for the operation. Transoesophageal echocardiography (TEE) is used in all patients to guide the CPB cannulation and to monitor heart and valve functions during the procedure. The patient is placed in a supine position on the operating table with the right hemithorax elevated at 20° and 2 defibrillator pads are placed across the chest wall.

#### Cannulation for CPB

After induction of the anaesthesia and intubation with a single lumen endotracheal tube, a TEE probe was placed for monitoring the heart and valve functions during the operation. Sterile washing, draping of the patient, and administration of heparin are done. Cannulation for CPB is then achieved through the common femoral vessels percutaneously in combination with the use of the MANTA^®^ (Essential Medical Inc., Malvern, Pennsylvania, PA, USA) vascular closure device (VCD) for femoral artery closure. Details of the use of the MANTA^®^ VCD were also previously published [7]. After successful puncture of the common femoral artery and vein, using the Seldinger technique and under TEE guidance, a wire is advanced into the descending aorta and superior vena cava respectively, followed by a skin and soft tissue dilator. For the arterial cannulation, an 8F puncture dilator is then inserted to evaluate the distance from the skin to the inner side of the femoral artery wall.

A 22- to 25-F femoral cannula (Bio-Medicus™ Multi-Stage Femoral Venous Cannulae, Medtronic, Minneapolis, MN, USA) is then inserted into the superior vena cava under TEE guidance with a bicaval view. Followed by the arterial cannula (Bio-Medicus™ Arterial Cannulae, Medtronic, Minneapolis, MN, USA) into the right external iliac artery through the right common femoral artery and then into the descending aorta. Depending on the patient’s body surface area (BSA), an 18F arterial cannula and a 22F venous cannula are used for BSA < 2 m^2^ and a 20F arterial cannula with a 25F venous cannula for BSA ≥ 2 m^2^.

#### RAMT access

Access for the RAMT is achieved 2-cm lateral to the sternal border at the third right intercostal space (ICS) through a 3- to 5-cm skin incision. The pleural space is then accessed laterally after dividing the intercostal tissue without resection or dislocation of the rib or cartilage junction, while special care is taken as to preserve the right internal thoracic vessels from damage. A soft tissue retractor (Valve Gate™ Soft Tissue Protector, Geister, Germany) is placed to optimize the exposure by helping to spread the chest wall tissues. The pericardium is then opened 2–3 cm above the phrenic nerve up to the innominate vein and caudally towards the inferior vena cava. In order to obtain an optimal position of the aorta and the aortic root, pericardial stay sutures are then placed.

Two additional small incisions are then performed to accommodate for the 3D camera port (Aesculap Einstein Vision, Tuttlingen, Germany) and the Chitwood aortic clamp (Scanlan International, Inc, St Paul, MN, USA). For the camera port, a 5-mm small incision was performed in the ICS above the main incision and a 5-mm incision laterally to the main incision in the same ICS for the aortic clamp. In order to lower the risk of air embolism, carbon dioxide is then infused through the camera port at a rate of 3 l/min. A long cardioplegia catheter (Medtronic DLP 9F, Ref 10012) in then inserted into the ascending aorta above the sinotubular junction and secured with a 3–0 polypropylene purse-string suture. This catheter is later used to vent the aortic root after the removal of the clamp. The left ventricular vent is inserted through the right upper pulmonary vein and is also secured with a 3–0 polypropylene purse-string suture.

Following the cross-clamping of the ascending aorta, crystalloid cardioplegia (Custodiol; Koehler Chemi, Alsbach-Haenlien, Germany) is administered in the aortic root in an antegrade fashion. In case of aortic regurgitation, cardioplegia is also delivered directly into the coronary ostia after cardiac arrest and aortotomy. This approach to cardioplegia was also employed in cases of mitral valve replacement surgery, as a concomitant aortotomy and aortic valve replacement was also performed in the presence of a significant accompanying aortic valve insufficiency.

Normothermic CPB is then achieved. It is important to assess the venous drainage and to obtain a satisfactory decompression of the right heart following the administration of cardioplegia before proceeding.

#### Replacement of the aortic valve

After cardiac arrest and emptying, a transverse incision of the ascending aorta is performed, followed by multiple 4–0 Prolene stay sutures in the aortic wall and aortic valve commissures to obtain a better valve exposure.

**Figure 3:**
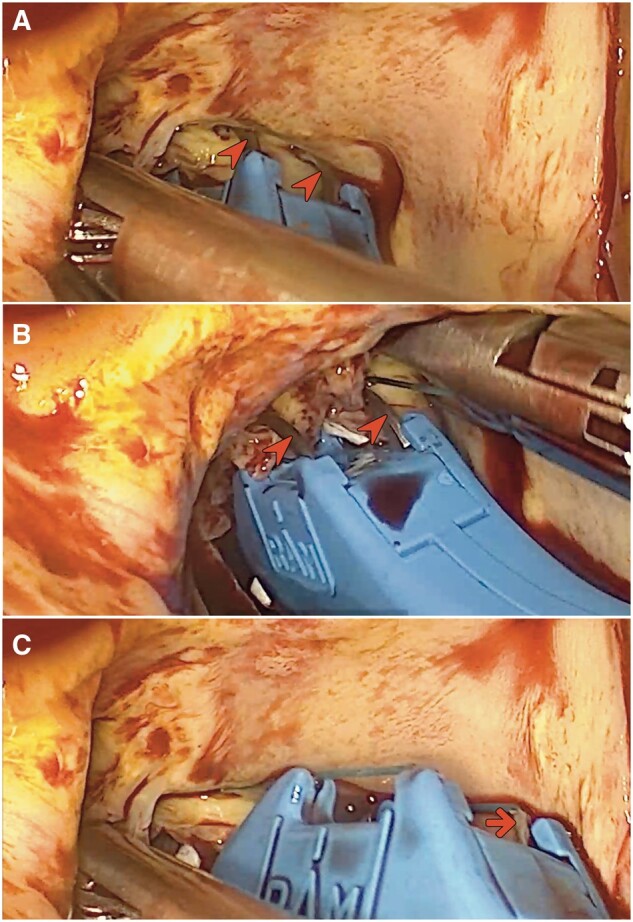
(**A** and **B**) When the lever is depressed, the 2 curved needles (arrowheads) extend from their housing in a fixed arc trajectory across the tissue gap and engage the needle caps of the Cor-Suture^®^ Quick Load^®^ suture. (**C**) Releasing the lever retracts the needles back through the tissue, attached to both suture needle caps (arrows).

The native aortic valve is entirely excised followed by the debridement of any residual calcified tissue from the annulus, which is then appropriately sized. Pledgeted mattress annular sutures (RAM^®^ Cor-Suture^®^ Quick Load^®^) are then placed using the RAM^®^ device starting with the base of the non-coronary cusp, followed by the left coronary cusp and finally the right (Fig. 3). Step-by-step use of the RAM^®^ device was previously published [5]. The sutures are then unloaded from the RAM^®^ device into a Sew-Easy^®^ Cassette Tip (Fig. 2C). The Sew-Easy^®^ device is then used to place both ends of the suture loop through the sewing cuff of the prosthetic valve (Fig. 1B) After seating the valve, the Cor-Knot^®^ (LSI Solutions) (Fig. 1A) is then used to fasten and secure the valve sutures.

Closing of the aortotomy is then performed in 2 layers with 4–0 Prolene, followed by de-airing the heart. While access to the anterior right wall of the right ventricle is still possible, ventricular pacing wires are placed and the cross-clamp removed. After weaning from CPB, protamine is administered at a 1:1 ratio to heparin. The correct function of the prosthesis is then assessed with TEE.

#### Replacement of the mitral valve

Access for mitral valve replacement is also obtained through RAMT endoscopically as detailed above at the level of the fourth ICS. This approach enables an excellent visualization of the inter-atrial groove and thus access to the mitral valve.

After starting normothermic CPB, cardiac arrest and subsequent emptying, the mitral valve is accessed through an incision of the interatrial groove. An atrial retractor (Valve Gate™ Holders Set Mitral, Geister, Germany) is then inserted and an anterior retraction is performed for a better visualization of the left heart cavities including the mitral valve. After excision of the mitral valve leaflets, or only the anterior leaflet in cases where the posterior mitral leaflet is preserved, the RAM^®^ device is used as described above to place pledgeted sutures through the posterior aspect of the mitral valve annulus, followed by the anterior aspect. Using the Sew-Easy^®^ system (cassette tip and device), the sutures are then passed through the corresponding part of the prosthetic sewing cuff. The valve can then be seated, and each suture is then fastened and secured using the Cor-Knot^®^ as described above. Once the valve is seated and secured, a water leak test is performed, and the left atrium is closed in 2 layers of continuous 4–0 Prolene sutures. De-airing is then achieved through the aortic vent catheter under TEE, which is also used to assess the prosthesis function and to check for PVLs.

#### Decannulation and closure

For CPB decannulation, venous hemostasis is achieved by applying a circular suture followed by the removal of the cannula and manual compression, and arterial hemostasis is achieved with the use of the MANTA^®^ VCD. A pleural drain is then inserted through the cross-clamp incision site and the ribs are secured with 2 FiberWire (Arthrex; Naples, FL, USA). The wound is finally closed in layers.

### Statistical analysis

All continuous variables were manually checked for normality using QQ-plots and the Shapiro–Wilk test, and accordingly represented as mean ± standard deviation or as median (interquartile range). Categorical variables were represented as absolute number and frequency.

The statistical analysis was performed using the *R* statistical software (R Foundation for Statistical Computing, Vienna, Austria).

### Follow-up

Clinical data from the initial hospitalization were prospectively entered into our institutional database. Discharged patients were contacted by mail or telephone to assess their clinical status ending in June 2023 and was 100% completed.

## RESULTS

Technical success was achieved in 100% of patients. Mitral-, aortic- and double valve surgery was performed in 54 (81.8%), 10 (15.2%) and 1 (1.5%) patients, respectively. Mean CPB time and AoX time were 97.9 ± 20.9 and 66 ± 15.7 min, respectively. Detailed procedural characteristics are shown in Table 2.

**Table 2:** Intraoperative outcomes

Outcome (total *N* = 66)	*N* (%)
Non-elective surgery	0 (0%)
Isolated aortic valve replacement	54 (81.8%)
Isolated mitral valve replacement	10 (15.2%)
Aortic + mitral valve replacement	1 (1.5%)
RAM size	
3.5	11 (16.7%)
5.0	55 (83.3%)
Aortic valve size	
21	8 (14.6%)^a^
23	23 (41.8%)^a^
25	21 (38.2%)^a^
27	3 (5.45%)^a^
Mitral valve size	
29	1 (9.1%)^a^
31	9 (81.8%)^a^
33	1 (9.1%)^a^
CPB (min), mean ± SD (overall)	97.9 ± 20.9
Aortic valve replacement	101 ± 20.4
Mitral valve replacement	83.7 ± 19.5
Doube valve replacement	116.1 ± 0
AoX (min), mean ± SD (overall)	66 ± 15.7
Aortic valve replacement	69.7 ± 15.4
Mitral valve replacement	51.2 ± 12.5
Doube valve replacement	74.2 ± 0
Concomitant procedures	17 (25.8%)
TVR	3 (4.6%)
LAA closure	5 (7.6%)
Cryoablation	6 (9.1%)
Morrow resection	6 (9.1%)
Aortic calcification removal	1 (1.5%)
Mitral calcification removal	8 (12.1%)

All values are represented as *N* (% of the total study population) unless otherwise specified.

a Value is represented as *N* (% of the total number within each valve type).

AoX: aortic cross-clamping time; CPB: cardiopulmonary bypass time; IQR: interquartile range; LAA: left atrial appendage; SD: standard deviation; TVR: tricuspid valve repair.

Postoperative outcomes are summarized in Table 3. Median ICU and hospital stays were 1 [1–2] and 9 [7–13] days, respectively. One conversion to sternotomy for bleeding was noted. One patient (1.5%) required PM implantation due to postoperative atrioventricular block. In-hospital mortality and 30-day mortality as well as the incidence of PVLs were zero. No wound healing disorders or cerebrovascular events were reported during the 30-day postoperative follow-up period.

**Table 3:** Postoperative outcomes

Outcome (*N* = 66)	*N* (%)
Stroke	1 (1.5%)
Delirium	0 (0%)
Intrathoracic bleeding requiring surgery	6 (9.1%)
Re-thoracotomy	5 (7.6%)
Conversion to sternotomy	1 (1.5%)
Low cardiac output syndrome	0 (0%)
Acute kidney injury	1 (1.5%)
Atrial fibrillation	11 (16.7%)
Atrio-ventricular block	7 (10.6%)
Temporary	6 (9.1%)
Requiring a pacemaker	1 (2%)
Pneumothorax	3 (4.6%)
Extra-corporeal membrane oxygenation	0 (0%)
Myocardial infarction	1 (1.5%)
Respiratory insufficiency	2 (3%)
Treated invasively	0 (0%)
Wound revision	0 (0%)
ICU stay (days), median [IQR]	1 [1–2]
Hospital stay (days), median [IQR]	9 [7–13]
Required transfusion of RBC	21 (41%)
0 transfusions	41 (62.1%)
1–2 transfusions	11 (16.7%)
3–4 transfusions	6 (9.1)
>4	8 (12.1%)
30-day mortality	0 (0%)
In-hospital mortality	0 (0%)

All values are represented in *N* (%) unless otherwise specified.

ICU: intensive care unit; IQR: interquartile range; RBC: red blood cell.

## DISCUSSION

Due to the steep learning curve and operative complexity of MIVS, including the increased difficulty of placing sutures, the technique has been proposed to be associated with increased operative times [8], of particular concern was the AoX time, which is an established risk factor for mortality and morbidity in both low and high risk patients [9, 10]. Aiming in widening the adoption of the minimally invasive techniques in valve surgeries, the RAM^®^ device is a novel technology introduced to facilitate the placement of valvular ring sutures. Our results show that aortic or mitral valve replacement surgeries aided with RAM^®^ are associated with favourable CPB and AoX times. However, the current literature lacks in data concerning the use of RAM^®^ in totally endoscopic minimally invasive valve replacement (TEMIVR) through RAMT except for a 5 patient initial experience series published by Wong *et al.* reporting an AoX average duration of 100 min [11]. Our own previously published CPB and AoX times for minimally invasive aortic valve replacement surgeries before the introduction of RAM^®^ use are lower than the results of the current series, with a mean cross-clamping time of 38 ± 12 min [6]. Those durations are however the result of years of cumulative experience with endoscopic minimally invasive techniques performed by a team of surgeons, anaesthetists, perfusionists and scrub nurses who developed an optimized, and effective communication between themselves. The current series describes however our first experiences with a novel device that requires an additional learning curve in itself, and we believe that with the optimization of the implementation of RAM^®^ within our surgical process, a further definitive decrease of cardiac ischaemic times is to be observed, as the team surpasses that initial learning curve. Nonetheless, our results still show low or comparable cross-clamping durations when compared to other studies reporting on MIVS through RAMT, endoscopic or otherwise [12–16].

Technical success was also shown to be favourable. The technical advantage of using the RAM^®^ device in endoscopic valve implantation is theoretically self-evident. By sparing the surgeon the need to manually implement the annular horizontal mattress sutures through a limited surgical access and with long instruments, the rapid and secure deployment of the successive threads using the automated suture placement device ensures a consistent quality as well as a constant size and distance between each individual suture. This is reflected by the results of our current series as technical success was achieved in all patients with no PVL noted throughout the study population. In combination with the low incidence of postoperative PM-dependent arrhythmias, our results highlight the advantage of using RAM^®^ associated with a conventional aortic valve instead of a sutureless valve, as the latter is associated with an increased incidence of PVLs and postoperative PM requirements, as well as increased costs with no advantage to conventionally sutured valves [17].

Although there is to our knowledge no other representative study on the RAM^®^ device, with regards to other post-operative morbidities as well as mortality, our results still show similar if not better outcomes when compared to studies reporting on aortic or mitral valve replacement surgeries through RAMT [16, 18]. Rates of re-exploration for intrathoracic bleeding in the current series was however relatively high with 6 patients (9.1%) requiring a return to the operating room. In all of those cases however, the source of bleeding was not found to be related to the use of the RAM^®^ device in itself. Additionally, the small sample size of the current series is consequently not representative of the population of patients undergoing TEMIVR. A larger matched study comparing the outcomes of using the RAM^®^ device versus manual annular suturing is however currently in development, which could potentially confirm whether these results are in fact the result the random effect of such a small sample-sized series.

TEMIVR offers several advantages when compared to conventional valve surgery through sternotomy or other minimally invasive techniques such as partial sternotomy [14, 16, 19, 20]. These include among others a better cosmesis, better pain profile, lower blood requirements and lower incidences of postoperative bleeding. It has however a steep learning curve and is technically challenging, hindering its wider adoption. Technologies such as the RAM^®^ device can help mitigate some of those technical challenges in facilitating the placement of the valvular ring sutures. Our current series shows that in addition to offering a technical ease of use, it is a safe and valuable addition to the arsenal of a surgeon experienced in minimally invasive techniques for valve surgeries.

### Limitations

Our current series is a single arm retrospective descriptive study with a limited population size, being an initial experience with the RAM^®^ device. Additionally, there are to our knowledge no other representative study on the device especially when used in combination with totally endoscopic MIVS, and thus, there are no other experience with this device that can be referred to in the literature.

Furthermore, MIVS is known to have a steep learning curve, however the surgeons operating on the patients of this series are experienced in TEMIVR, which could explain the favourable results observed herein. Additionally the majority of patients included were retrospectively shown to be relatively healthy with lower age, body mass index, Euroscore II scores, as well as left ventricular function, these parameters were however not considered in the selection process for the use of the RAM^®^ device, which was rather guided by each surgeon’s preference and experience with the device itself. As this series serves as a first experience report evaluating the safety and feasibility of using the RAM^®^ device in TEMIVR, a larger matched comparative study is needed to draw conclusions regarding its efficacy, which is currently in the writing process.

## CONCLUSION

The usage of RAM^®^ device is a safe, feasible and effective approach to the implantation of aortic or mitral valves in totally endoscopic minimally invasive valve surgeries through RAMT and yield excellent early outcomes. Larger sized studies over an extended period of time are needed to evaluate the effect of using RAM^®^ on operative times, especially AoX duration.

## Data Availability

The data underlying this article will be shared on reasonable request to the corresponding author.

## ABBREVIATIONS

AoX: Aortic cross-clamping
BSA: Body surface area
CPB: Cardiopulmonary bypass
ICS: Intercostal space
ICU: Intensive care unit
MIVS: Minimally invasive valve surgery
PM: Pacemaker
PVL: Paravalvular leak
RAMT: Right anterior mini-thoracotomy
TEE: Transoesophageal echocardiography
TEMIVR: Totally endoscopic minimally invasive valve replacement
VCD: Vascular closure device
